# Manipulation of Axonal Outgrowth via Exogenous Low Forces

**DOI:** 10.3390/ijms21218009

**Published:** 2020-10-28

**Authors:** Sara De Vincentiis, Alessandro Falconieri, Vincenzo Scribano, Samuele Ghignoli, Vittoria Raffa

**Affiliations:** Department of Biology, University of Pisa, SS12 Abetone e Brennero 4, 56127 Pisa, Italy; sara.devincentiis@phd.unipi.it (S.D.V.); alessandro.falconieri@biologia.unipi.it (A.F.); v.scribano@studenti.unipi.it (V.S.); s.ghignoli@studenti.unipi.it (S.G.)

**Keywords:** axon outgrowth, mechanical force, therapy

## Abstract

Neurons are mechanosensitive cells. The role of mechanical force in the process of neurite initiation, elongation and sprouting; nerve fasciculation; and neuron maturation continues to attract considerable interest among scientists. Force is an endogenous signal that stimulates all these processes in vivo. The axon is able to sense force, generate force and, ultimately, transduce the force in a signal for growth. This opens up fascinating scenarios. How are forces generated and sensed in vivo? Which molecular mechanisms are responsible for this mechanotransduction signal? Can we exploit exogenously applied forces to mimic and control this process? How can these extremely low forces be generated in vivo in a non-invasive manner? Can these methodologies for force generation be used in regenerative therapies? This review addresses these questions, providing a general overview of current knowledge on the applications of exogenous forces to manipulate axonal outgrowth, with a special focus on forces whose magnitude is similar to those generated in vivo. We also review the principal methodologies for applying these forces, providing new inspiration and insights into the potential of this approach for future regenerative therapies.

## 1. Introduction

Axonal elongation, guidance and navigation are crucial for the development of the nervous system. They require motion, and Newton’s laws states that motion is caused by forces. Neurobiologists have learned how axons generate forces and, most interestingly, that axonal outgrowth can be driven by force application only [1,2,3]. The endogenous mechanisms of force generation have been extensively studied and reviewed in the last decades, while the literature still lacks points of view regarding the potential use of exogenous forces of strength and magnitude similar to the endogenous ones. Although force is perhaps the strongest inducer of axonal outgrowth [4], no practical applications have yet been proposed. One reason why the therapeutic potential of force as a regulator of axonal outgrowth has been neglected for decades is the lack of methodologies to translate research outcomes into clinical research/practice. Material sciences and nanobiotechnology hold promise for novel methods for exploiting exogenous forces in regenerative therapies.

Here, we intend to provide an overview of the various methods to manipulate axon outgrowth via exogenous forces, showing the ability to modulate every phase of neuronal growth and highlighting any future therapeutic applications. This review begins by describing how axons perceive mechanical stimuli from the environment and convert them into a signal generating the intracellular force that is responsible for motion. Inspired by Weiss’s visionary hypothesis that the growth of an animal’s body places axons in a condition of stretching that could be responsible for axonal growth following synaptogenesis [5], here, we focus on the effects of prolonged exposure to low magnitude forces, more similar to those naturally occurring in vivo. We reveal how exogenous mechanical forces act on axons, possibly resulting in the activation of pathways that influence every step of neuron development, from neurogenesis to synaptogenesis. Finally, we review the methodologies for force application in terms of their therapeutic potential to stimulate axonal outgrowth in regeneration therapies.

## 2. Endogenous Force Generation in Axonal Outgrowth

Force is required to generate motion, and a force requires energy. It is thus not surprising that the axon has a complex machinery to generate force, which is one of the key regulators of axonal outgrowth. This section describes the mechanisms behind the generation of the endogenous force and the force against the extracellular matrix to produce a motion.

### 2.1. Axon: The Neuron Machinery That Moves Forward

Neurons are highly polarized cells that are able to extend axons, which then grow for long distances to reach the target during the process of development. The growth cone (GC) is the apical structure at the end of the axon that drives the axonal extension. This process leads to the axon elongation and guidance, which is defined as the increase or addition of new bulk material at the leading edge.

For decades, it was thought that the new material is added at the GC while the remaining part of the axonal cytoskeleton stays stationary, but recent work has contradicted this view [6]. Axonal transport is clearly essential for mass addition. It is driven by molecular motors that run on microtubules (MTs), the main cytoskeletal “tracks” for transportation. It is made up of retrograde and anterograde transport for the movement of all the components required for the correct functioning of the neuron. It also accounts for the transport of the matter needed for axon growth.

### 2.2. The Contractile Force Generated at the GC Is Responsible for the Movement of the GC

**Figure 1:**
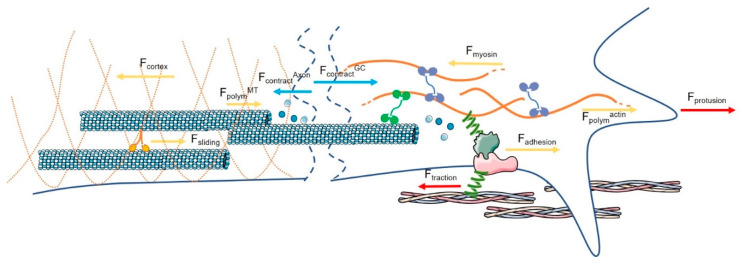
The endogenous forces of the axonal (on the left) and growth cone (GC) compartment (on the right). In the axon, MT polymerization pushes against the GC (F_polym_^MT^), and the sliding of molecular motors (yellow/orange proteins) creates an additional pushing force (F_sliding_). However, the axonal shaft generates a contractile force that pulls the GC (F_contract_^Axon^), above all generated by the cortex force (F_cortex_) of the actin, which antagonizes the pushing forces. In the GC, actin filament (orange lines) polymerization generates a force that pushes on the membrane (F_polym_^actin^). NMII (violet proteins) powers the actin RF, generating an opposite F_myosin_. When molecular clutches engage the F-actin filaments, they oppose this force and stretch (F_adhesion_). A traction force F_traction_ is thus transmitted to the substrate. Consequently, the RF slows down, and actin polymerization/de-polymerization rates are not balanced. This creates a force that pushes the apical structure forward (F_protrusion_) as well as a force that pulls the axon shaft (F_contract_^GC^).

The GC generates contractile forces that pull the axon shaft [7,8]. The cytoskeleton then generates and transmits this force. Both the axon shaft and GC are rich in cytoskeletal elements, such as actin filaments and MTs, in varying proportions. There are three zones in the GC with different cytoskeletal compositions: (i) a central domain (C) where stable MTs and organelles spill out of the axon shaft; (ii) a peripheral domain (P) rich in a network of actin filaments whose polymerization creates the protrusions for invading and exploring the external space; and (iii) a transition zone (T) between these two regions, where the translocation of MTs from the C domain and the flow of actin from the P domain are dynamically coupled [9,10]. Actin filaments de-polymerize in the T domain and polymerize in the P domain, pushing on the membrane with their growing (+) end (Figure 1) [10]. Membrane tension opposes this pushing force, and a retrograde flow (RF) of actin is generated, powered by non-muscle myosin II (NMII), which recycles actin filaments to the T domains, where they de-polymerize (Figure 1). A balanced situation between actin RF and actin polymerization leads to no elongation in the steady state condition [8,11].

The dynamic association between extracellular matrix (ECM)-bound integrins and the force-generating actomyosin cytoskeleton constitutes that mechanical connection, namely, of molecular clutches [12]. According to the adhesion clutch hypothesis [13], adhesion at the GC, which is regulated by a multitude of physical and chemical signals [14], creates a physical coupling between the F-actin filaments and the molecular clutches. When molecular clutches engage the F-actin filaments, they resist the force generated by NMII and slow down the actin RF. The molecular clutches are thus stretched, which, in turn, generates a traction force onto the matrix at the adhesion point (Figure 1). In the end, the actin polymerization/de-polymerization rates are no longer balanced, thus generating a force of several piconewtons (pN) that pushes the filopodial and lamellipodial edge forward [15]. Interestingly, as clutches are dynamic structures, spatial and temporal tension fluctuations can be observed in the GC due to the formation and disassembly of adhesions [16]. Contrary to what was originally thought, MTs are not passive contributors to this process. In the steady state, MTs explore the T domain by moving back (by coupling to the RF, movement powered by NMII) and forward (by assembly, movement powered by dynein). During the initial (latent) phase of elongation, some highly dynamic MTs spend more time at the adhesion sites, uncoupling from the actin RF [17]. Later, when the RF strongly attenuates and traction force increases at the adhesion site, Rho-dependent actomyosin contractility is responsible for massive MT translocation through association with actin bundles (C domain) and actin arcs (T zone) [18], while in the P domain, MTs simply advance towards the adhesion site because of actin RF attenuation and actin clearance [19].

Indeed, the myosin-dependent contractility is responsible for generating a contractile stress that is constant, but force fluctuations might result from the dynamics of adhesion assembly and disassembly, eventually providing a mechanism for probing the ECM [16]. The contractile force generated by NMII is relatively high: a single myosin protein is able to exert around 1 pN; there are an estimated 1000 myosin proteins per µm in the P zone, and the resulting contractile force is about 1 nN µm*^−^*^1^ [8]. The contractile force generated in the *Aplysia* GCs has been estimated to be about 2 nN [7].

### 2.3. The Contractile Force Generated at the Axon Level Influences the Bulk Translocation

The axon also generates a contractile force that pulls the GC (Figure 1). The main components of the axonal cytoskeleton are the axonal actin cortex underneath the plasma membrane and the MTs, which are relatively stiff and organized into dense bundles cross-linked by MT-associated proteins (MAPs). The axonal actin cortex and NMII-driven contraction produce circumferential and longitudinal contractile forces along the axon (Figure 1) [19]. MT assembly generates a compressional force that pushes against the GC. However, a growing body of evidence indicates when the molecular motors residing in MT bundles slide, this exerts an additional mechanical force (Figure 1) [20].

Motor proteins are also able to move the cytoskeletal elements, creating a necessary connection between endogenous force generation and mass translocation [21]. Dynein exerts forces between microfilaments and MTs to push forward the cytoskeletal meshwork as a single structure [21,22]. Kinesin-1 belongs to unipolar motors that have one domain, called a walking domain, which binds the MTs, and another domain that loads the cargo (or interacts with another filament). The motors create the force to drive the MT bundle expansion. Kinesin-5 belongs to bipolar motors, which have two walking domains that slow down the relative motion between two MT parallel filaments [23]. Dynein and kinesin-1 promote the axonal outgrowth with the generation of endogenous low forces and bulk translocation; instead, kinesin-5 slows down the process with an inhibitory effect [21,22,23,24]. The range of these forces generally varies between 4 and 8 pN when generated by a single motor protein, and up to 9 pN when they act as multiple-protein motors or in teams [25]. The sum of these opposing forces along the axon results in a net contractile force that is relatively low. In the *Aplysia* model, the contractile force generated in the axons has been estimated to be about 0.6 nN [7].

### 2.4. Variations of Endogenous Force Generation Modulate Axon Growth

The strong contractile forces generated in the GC and the weaker contractile forces along the axon regulate axonal growth [9]. Cytoskeletal dynamics and motor proteins are key actors in this process. Endogenous forces generated by kinesins or dynein along the axon slow down or promote the bulk translocation at the GC. On the other hand, endogenous forces at the GC, which are generated by acto-myosin contraction, drive the structure forwards [21,22,23,24].

The level of the endogenous force generated also depends on the substrate. Some studies appear to contradict each other and axon elongation/guidance seems to be influenced in different ways by substrate stiffness, depending on the cell type and the experimental conditions [26]. The theoretical explanation is that there are at least two mechanisms contributing to the traction force generation. Once the clutches bind to the substrate, it is deformed and the traction force increases. Soft substrates have a higher deformation and a slower force loading than stiff substrates. Above a rigidity threshold, force loading can lead to either clutch disengagement or talin unfolding, which have opposite effects [27,28]. When force loading is too fast, clutches rapidly reach their breaking strength and disengage from the substrate before other clutches have time to bind, thus reducing the traction force. On the other hand, the force loading causes talin unfolding, and the exposure of a vinculin binding site leads to adhesion reinforcement and adhesion maturation.

The adhesion site is where several mechanosensitive (MS) proteins of the molecular clutches are concentrated. Their stretching often exposes cryptic domains, which regulate the clutching. As in non-neuronal cells, adhesion contact points appear to require integrin engagement. The substrate triggers talin-dependent integrin activation, leading to the recruitment of scaffold proteins that link the clutch to actin RF [29]. Talin has itself an actin-binding domain, but its binding partner vinculin, which is recruited by talin when force is generated [30], may play a prominent role in reinforcing integrin–actin linkages [31]. Vinculin likely anchors p130Cas, a protein that has a central substrate domain, which is intrinsically disordered. The vinculin recruitment of p130Cas in a focal adhesion (FA) allows this central domain to stretch, thereby making tyrosine motifs accessible to Src kinases for phosphorylation, initiating several cascade pathways [32]. Filamin, another FA protein, has an amino-terminal actin-binding domain, followed by an integrin-binding domain, which is normally folded. However, when force is applied, this intracellular interaction is released, increasing the level of filamin binding to integrins [33] and offering binding sites for several signaling molecules such as Rho, Rho-associated protein kinase (ROCK), protein kinase C (PKC) and p-21 activated kinase (PAK) [34]. Other scaffold MS proteins include focal adhesion kinase (FAK) and protein-tyrosine phosphatase alpha (RPTP-alpha). FAK is essential for dynamic adhesion (assembly/disassembly) and axon guidance [35]. RPTP-alpha senses a stiff substrate, promoting Src- and Cas-mediated adhesion complex strengthening [36]. Force exertion on mature adhesions also triggers Rho GPTase activity, which results in myosin II assembly into filaments, promoting the interaction of myosin II with actin filaments and acto-myosin contraction.

Along the axon, the adhesion on the substrate also plays a role. When there is a strong adhesion, forces are dissipated, and bulk transport diminishes. When the interaction between the axon and the substrate is stronger, most of the transport is near the GC [37]. Several MAPs also play a part in the equilibrium between the forces that oppose the axons, i.e., the expansion force generated by MTs/motor proteins and the contractile force generated by the acto-myosin structure. One protein is tau, which inhibits the forces made by each type of kinesin motor in order to facilitate dynein motility. Thanks to tau, dynein is able to generate higher pushing forces to increase the axonal elongation [22,38].

The axons are also rich in membrane receptors, especially at the GC [39]. A lot of molecules have been found to modulate the generation of the endogenous force. Many signaling molecules exploit contact adhesion to regulate clutching directly. Netrin-1 converts chemical signaling into force by activating its receptor (DDC) and its downstream effectors (Cdc42 and Rac1), which trigger PAK-mediated shootin1 phosphorylation, promoting shootin1–actin interaction, cluster engagement, a reduction in RF, the generation of force and axon growth [40]. Nerve growth factor (NGF) signaling influences the speed and the direction of the axon growth of dorsal root ganglia (DRG) neurons grown on laminin-1 by slowing actin RF [41].

Other signaling molecules, such as brain-derived neurotrophic factor (BDNF) [42] and semaphorins [43], also regulate axonal guidance by asymmetrically modulating the generation of mechanical force at the level of adhesion points/molecular clutches. The gradients of attractive or repulsive guidance cues induce the asymmetrical activation of the translation of proteins that build up or disassemble the cytoskeleton, respectively, leading to axon steering [44,45]. In this context, it is important to mention the key role played by MS ion channels that strongly regulate GC motility [14], but there are also ion channels not conventionally described as mechanosensitive that can open in response to force [46]. Many of these channels regulate axon motility and pathfinding through the direct control of Ca^2+^ and are modulated by cell–substratum interactions [47]. One mechanism by which filopodial Ca^2+^ transients can regulate GC motility and guidance is through the local activation of calpain [48,49]. Calpain is a protease that can cleave certain enzymes (e.g., the calpain-catalyzed activation of PKC) and numerous adhesion and actin-binding proteins [50], modulating or disrupting mature FAs [49]. Ca^2+^ channels have been found to localize near integrin adhesion sites and Ca^2+^ signals in the areas of higher traction forces [47], suggesting that substrate rigidity can modulate the Ca^2+^ channel response. This contribution of Ca^2+^ signals could partially explain the apparent discrepancies in the literature related to the influence of the substrate stiffness. This hypothesis could provide an explanation for those studies that have reported a biphasic behavior, which consists of a linear increase in the traction force with increasing substrate stiffness until reaching a plateau at a sufficiently high value of rigidity [51].

Recent studies established that the cytoskeleton, molecular motors, cell adhesion molecules (CAMs) and the extracellular matrix are also involved in the latest processes of axonal growth. Due to the increased complexity of mature neurons compared to developing axons, much less is known about the mechanical aspects of synaptogenesis and plasticity, but a complete review about the current knowledge is given by Kilinc [52].

## 3. Exogenous Low Forces Stimulate Axonal Outgrowth

**Figure 2:**
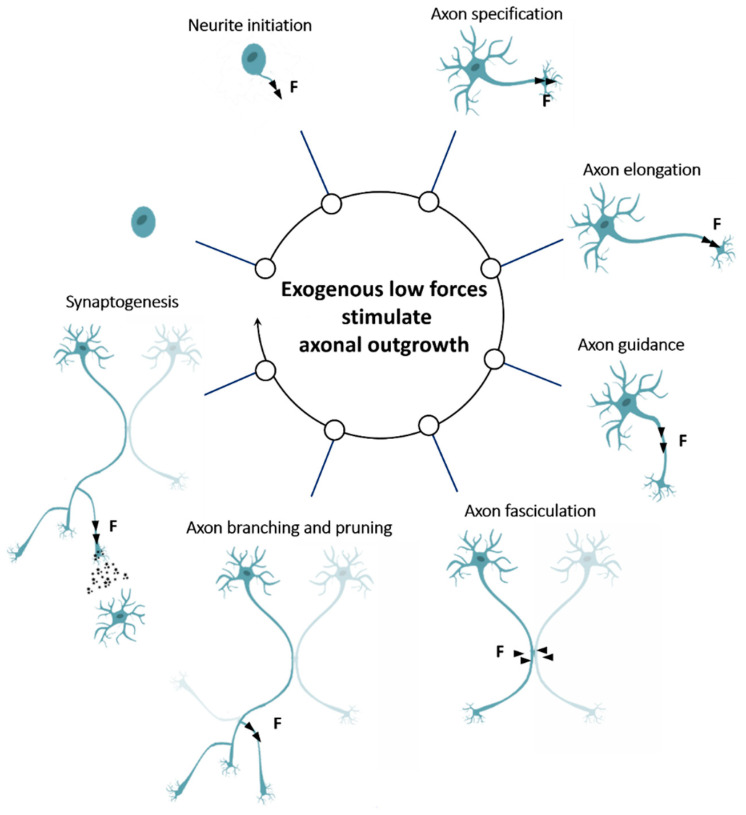
Mechanical force influences every phase of neuronal growth.

As the axon has machinery to sense and generate forces, it also responds to the application of exogenous forces. Neurons are mechanosensitive over three distinct ranges of force magnitude (for a review, see [53,54]); however, in this review, we will focus on forces whose magnitude is similar to those generated in vivo (<2 nN) [7,55]. Nevertheless, through these exogenous low forces, it is possible to influence every phase of neuron development (Figure 2).

### 3.1. Exogenous Force Promotes Neurite Initiation

Shortly after plating, immature neurons become attached to the matrix. Neurons then establish several short processes (called “neurites”), the process referred to as neurite initiation [56]. In the 1980s, pioneer studies using DRG neurons from 10–12-day-old chick embryos cultured in glass coverslips showed that the mechanical tension leads per se to axon initiation [57]. Subsequently, experiments have shown that tension applied above a force threshold can break the symmetry in several types of cultured neurons by initiating neurites de novo from a rounded cell body, irrespectively of the method of applying force [58,59,60,61]. The forces typically required for inducing neurite initiation are in the range of 0.3–10 nN [58]. With the application of these forces, initiated neurites developed GCs capable of normal motility and axonal elongation. Such neurites also contained a normal array of MTs as assessed by immunofluorescence and by electron microscopy [61]. In 2003, Fass and Odde showed that neurite initiation from embryonic chick forebrain neurons was a first-order random process, whose rate increased with increasing force [62]. Recently, Magdesian et al. partially contradicted these results by revealing that the process of neurite initiation is necessarily linked to the process of new mass addition, and pulling neurites at a rate faster than 0.5 µm min*^−^*^1^ during the first 5 µm results in neurite breaking [63].

### 3.2. Exogenous Force Promotes Axon Specification

After the initiation phase, neurites undergo rapid growth and retraction cycles. At this stage, neurons still appear to be in the unpolarized state, since the neurites are apparently identical morphologically or immunocytochemically, and it is unclear which one(s) will become (an) axon(s) or dendrite(s) [56]. Within 24 h, polarity first becomes evident, as one of the immature neurites shows enhanced elongation and acquires axonal characteristics, the process referred to as “axon specification” [64].

Lamoureux et al. showed that external mechanical tension can stimulate the differentiation of minor processes of rat hippocampal neurons into axons [65]. Subsequently, the developmental course of experimental neurites was found to be similar to that of unmanipulated spontaneous axons, as well as the presence of the same molecular markers being found. Kunze et al. investigated what was behind this observation and revealed that the localized mechanical stimuli impact the polarity of cortical neurons by affecting the intracellular distribution of the cytoskeletal protein Tau [66], an important protein involved in the development of neuronal polarity [67]. The complete role played by mechanical signals in mediating single-cell polarity, however, remains unclear, although the effect on axon specification has been confirmed.

Once established, polarity is not rigid but instead is reversible, and mechanical tension also influences this reversibility. In a neuron typically extending only one axon, tension could stimulate the formation of multiple axons [65]. The hypothesis is that while neurite growth is usually inhibited in minor neurites after axon specification, this inhibition can be relieved by applying mechanical force. This presumably drives the redistribution of polarity effectors and, in turn, the induction of a second axon or multiple axons [68].

### 3.3. Exogenous Force Promotes Axon Elongation

For the first 2–3 days in culture, the other neurites remain quiescent and undergo little net elongation, while the axon continues to grow without retraction, the phase known as axonal elongation [56]. In 1984, Bray demonstrated that mechanical tension can also stimulate this process. The experiments, lasting about 30 min, showed the formation of bundles of neurites of a length equal to or greater than 300 μm when mechanical tension was applied along the major axis. Strikingly, the axons had not only increased length, when subjected to elongation, but also increased volume, while maintaining a normal ultrastructure and longitudinally aligned MTs and neurofilaments. In fact, neurons are able to sense the mechanical tension and respond by stimulating the addition of new proteins, membranes and cytoskeletal components [57]. Many variations of Bray’s pioneering experiments have been carried out by other groups, confirming previous findings [59,61,69,70,71,72,73]. Overall, regardless of the neuronal type, all neurons grow when subjected to external tension, with the elongation rate being directly proportional to the magnitude of tension applied [74]. Experimentally, applied mechanical tension can cause far more robust axonal growth than is observed “physiologically”, either in vitro or in situ. The most extreme example is the work of Smith and Pfister [4,75]. Axons of DRG neurons were subjected to a strain, showing elongation from 100 μm to 10 cm in two weeks, with an elongation rate of approximately 300 μm h*^−^*^1^ (i.e., 8 mm day*^−^*^1^), almost 10-fold greater than the typical movement rate of GCs, which is approximately 1 mm day*^−^*^1^. However, Smith and Pfister found some restrictions in the process of elongation. They showed that the elongation rate is linked to the rate of mass addition. In fact, they experienced the following limitations: the strain cannot exceed about 2% of the initial axon length, and a minimum time for conditioning is required, otherwise axons break. Under these conditions, the stretched neurons elongated but did not show a decrease in their caliber following ultrastructural analysis [75] and were functionally normal from an electrophysiological point of view [76]. The same result was obtained by Steketee and colleagues for postnatal axons from retinal ganglion cells (RGC) [77].

In the last two decades, various thresholds have been identified for elongation, depending on the methods used for applying the force. A force threshold for elongation of approximately 1 nN has been reported for neurites of PC12 cells [58] and for chick sensory neurons [61] elongated by the pulling force generated by glass microneedles; 15–100 pN was reported for the neurites of chick forebrain neurons [62] elongated by the magnetic force induced by magnetic microbeads. According to these studies, axonal elongation takes place above the threshold, but below there is only a viscoelastic deformation or retraction. Recent studies have contradicted this conclusion, pointing out that there is no threshold for elongation. Neurites of PC12 cells or hippocampal neurons stretched with forces in the range of 1–10 pN increased their length from 50 to 100% compared to the condition of spontaneous elongation, in 48 h [78,79]. Interestingly, the elongation rate was very similar to the one calculated in previous studies (0.1–1 µm h^−1^ pN^−1^) [61], although the applied force was five orders of magnitude lower than that used in previous work. A possible explanation for this discrepancy is that axons respond to extremely low forces acting for days as viscoelastic fluid, while neurites show an elastic behavior when subjected to intense forces acting for a shorter time [79]. Indeed, a similar result was obtained by Abraham et al. investigating the response of primary cortical neurons to cyclic strain over hours with physiologically relevant amplitudes and repeated frequencies [80]. Therefore, from recent experimental data, long-acting and low magnitude force significantly influences the outgrowth process [72,81,82,83,84].

### 3.4. Exogenous Force Promotes Axon Guidance

The next phase, in vitro, is the pathfinding towards the synaptic target. The GC of an elongating axon possesses detectors of guidance cues that translate environmental cues into directional movement and thus guide neuronal processes toward their destination [10]. It is now accepted that the application of exogenous forces may also influence neurite orientation. Initially, it was observed that neurite initiation and elongation always occurred along the axis of force application, consistent with the idea that the direct transmission of force through the membrane provides a directional cue for outgrowth [85]. In the study by Wu et al., a shear stress of 0.17 pN, generated with beads manipulated by optical traps, was used to turn the GC of individual axons in response to the shear [86]. Similarly, our research group demonstrated that a force below 1 pN can influence the orientation of PC12 cell neurites [87]. Re-positioning the neurites of single cortical neurons was found to have an optimal force range of 4.5–70 pN [66]. Abraham et al. found a dependency in the neurite orientation relative to cyclic strain. They found significant remodeling of the MT cytoskeleton, adaptation to the cyclic strain, and MT formation in the stretch direction [80].

### 3.5. Exogenous Force Promotes Axon Fasciculation

Fasciculation depends on molecular interactions between proteins in axonal membranes (axolemmas), which can promote fasciculation, defasciculation (i.e., an axon leaving a bundle of fasciculated axons) or GC repulsion [88]. Smit et al. revealed a new role of mechanical tension, which also helps regulate this process, through the control of axon shaft zippering [89]. Using the explant of mouse olfactory epithelium, they showed that axon zippering is regulated by a competition between two principal forces, axon–axon adhesion and mechanical tension, which tends to promote unzippering. Further studies are needed to fully understand the role of axonal tension in this process. The main effect at the cellular level has, to date, been observed by Katiyar et al., where the application of mechanical tension resulted in increased motor axon fasciculation. They used a novel combination of microtissue engineering and mechanically assisted growth techniques to generate healthy motoneurons projecting dense and fasciculated axonal tracts [81].

### 3.6. Exogenous Force Promotes Axon Branching and Pruning

Two important mechanisms take part in shaping the connectivity diagram of neuronal systems: axonal branching and pruning. Axonal branching is crucial for connecting a single neuron with multiple targets and therefore is essential for forming synapses. On the other hand, axonal pruning is the elimination by means of retraction or degeneration of excess or inappropriate axon branches [90,91]. Bray reported that tension plays a role in promoting branching [57,92], and our team recently also observed the same effect with primary hippocampal neurons. By applying forces below 10 pN on the whole axon for 2 days, we found a stimulation of axonal branching, with an increase in the number of both secondary and tertiary processes [78]. Detailed analyses of branch formation have revealed that structures largely depend on actin polymerization at early stages, while MT formation within these branches takes place at later time points [93,94,95]. However, Abraham et al. argue that MT networks are already formed within side branches, suggesting that the exogenous force might locally destabilize MTs and trigger the increase in branch formation [80].

It has been suggested that tension may act as a signal for axonal branch survival [70,96]. However, an increase in tension along one branch may not only lead to its stabilization, but also cause the retraction or elimination of axon collaterals [69,96]. It has long been considered that pruning is mainly regulated by an activity-dependent mechanism. Anava et al. provided direct evidence of the role that mechanical tension plays in axonal pruning, which leads to a step that precedes the known scheme of the activity-dependent stabilization of synaptic specificity [96]. They found that tension applied to invertebrate neurons promotes the stabilization of one set of axon branches while causing the retraction or elimination of axon collaterals. This can happen when the mechanical stress applied to the leading edges of the GC exceeds a threshold [97]. The process triggers a local, dramatic increase in calcium concentration, GC collapse, and the loss of the adhesion sites with the substrate; the neurite retracts and resembles a relaxing elastic coil spring. Finally, if the process is not completely withdrawn, a new GC is established, adhering to the substrate. Generally, the neurite starts re-growing in a new direction—away from the suprathreshold mechanical contact [97]. Together, these results suggest that the application of tension and the subsequent selection of some axonal arbors over others could be used as an instrument for shaping the morphology of the neurons and the network.

### 3.7. Exogenous Force Promotes Synaptogenesis

Once the GC has reached its target cell, it arborizes and establishes synaptic connections, forming functional neural networks [98]. Mechanical tension along neurites affects various aspects of this process such as synaptic vesicle dynamics [99,100], synaptic transmission [101], excitability [102] and network formation [63].

A key step during synaptogenesis is the accumulation of synaptic vesicles at the presynaptic terminal. Siechen et al. revealed that this vesicle accumulation at the presynaptic terminal is dependent on mechanical tension. In a model of the axotomy of fly motor neurons, presynaptic clustering was restored by mechanically pulling the severed axon, confirming tension dependency: the axon subjected to strain (5% of initial length) for 30 min showed 200% increased vesicle clustering. Siechen’s study speculates that stretch-dependent actin polymerization may create an actin scaffold that facilitates the accumulation of vesicles [100]. Ahmed et al. observed a similar qualitative response, demonstrating that mechanical stretching affects vesicle dynamics at a local and global scale of synaptic vesicle accumulation at the synapse [99]. The underlying molecular mechanisms that link mechanical tension and vesicle dynamics need further investigation, but these studies support the evidence that tension plays a role in triggering synaptogenesis [103].

Exogenous forces can also influence synaptic functions, through the modulation of neuronal communication. Some studies have shown an immediate and reversible increase in both spontaneous and evoked neurotransmitter release due to stretching in frog neurons. Interestingly, they found that the stretch enhancement of neurotransmission bypassed the usual Ca^2+^ triggering step in vesicle fusion: the release was reduced but still occurred [101,104,105,106]. They concluded that there is direct mechanical modulation of the release pathway [101]; thus, the process can be influenced by applying exogenous forces.

Fan et al. showed that synaptic excitability can also be regulated by an externally applied stretch. They found that by maintaining axons under low stretching for 10 min, they were able to increase the probability of a neuronal response [102]. Interestingly, excitability increased after every cycle of stretching. Thus, it seems that the slice “remembers” its past history of stretch, and its current excitability results from a cumulative effect of its past stretches [102]. Our research team obtained a similar result when testing the effect of stretching on primary hippocampal neurons. Stretched neurons showed, at 7 days in vitro (DIV7), a significant increase in the frequency (i.e., shorter inter-event intervals), but not in the amplitude, of spontaneous excitatory postsynaptic currents (sEPSCs). At DIV14, both the frequency and amplitude of sEPSCs were significantly higher than control cultures [78]. Fan et al. hypothesized that the increased clustering of synaptic vesicles previously reported [99,100] can lead to a higher docking ratio, more frequent spontaneous release and larger release upon stimulation [107]. This could explain the higher excitability upon completion of a stretch-baseline cycle [102], but it is still not clear what it is behind the cellular effect registered.

Magdesian et al. showed that force can be used to (re)wire neuronal networks. Pulling an axon or dendrite can trigger a new secondary process, which can be mechanically guided to form new synapses in less than one hour. Specifically, they elongated the new neurite for 60 µm until putting it in contact with the next bundle of axons and dendrites. After 30 min, a stable connection was formed. They showed that it is possible to create and control new functional neuronal connections; however, more studies are needed to understand whether and how micromanipulated connections differ from natural ones [63].

## 4. Local and Molecular Effects Triggered by Exogenous Low Forces

This section focuses on the effects of mechanical force at the molecular level, exploring the consequences of mechanical stimulation for fundamental neuronal mechanisms, such as cytoskeletal dynamics, intracellular calcium homeostasis, axonal transport, and molecular pathways activated by external tension. We also analyze the complex cross-talk between mechanical stimulation and chemical cues.

### 4.1. The Birth of “Stretch-Growth”

The role of force as an inducer of axonal elongation was first identified in 1941 when Paul Weiss called ‘‘towed growth’’ the mechanism by which neurons are forced to grow while being “drawn out” due to growth of the animal body [5]. In 2004, Smith et al. [75] coined the term “stretch-growth” (SG), which refers to the role of mechanical force in any phase of axonal outgrowth. The experiments performed in the last two decades have shed light on the role of tension in axonal outgrowth, providing a more general understanding of the underlying molecular mechanisms but with sometimes contradictory interpretations.

Only the axial component oriented from the axon hillock to the tip is productive for stretch-growth [79]. Stretch-growth requires continuous loading, and, within a few minutes after force removal, neurites resume the behavior of tip-growth [79,82]. In order to initiate neurites de novo, a force threshold needs to be overcome, which ranges from hundreds of piconewtons to a few nanonewtons, depending on the cellular model [54,55,56,58,59,60]. Conversely, the elongation rate was 0.1–0.3 µm h*^−^*^1^ pN*^−^*^1^, regardless of the force magnitude, which varied from 1 pN to tens of nanonewtons in different studies [58,59,60,61]. In fact, any extremely low force seems to induce elongation provided that it is applied for a sufficient time [79]. However, the upper force that can be applied to the neurite without causing disconnection is obviously constrained to the rate of new mass addition. Axonal material can travel from the cell body to the tip and vice versa by fast or slow axonal transport. Axon outgrowth is commonly regarded as being limited by the slowest component (neurofilaments and MTs) of the slow axonal transport. In fact, the axon elongation rate cannot exceed the rate of the slowest component of axonal transport. It moves at an average speed of 0.3–3 mm day^−1^, which is likely to be the consequence of a “stop and go” transport [108,109]. Consequently, the application of high forces over a long time would cause axon breaking, except for the application of a duty cycle consisting of a stretching time spaced out by a minimum time for conditioning [75]. Under these conditions, axon caliber does not change or may sometimes increase [75], and the big question for scientists in the last decade was how new mass is added along the length of the axon to prevent thinning. In 2011, Suter and Miller proposed the “stretch-growth model”, which postulates the “intercalated mass addition”, meaning that new mass is added at any point along the axon where tension is perceived [1]. This is in opposition to the “tip-growth model”, which considers the GC as the exclusive site where new mass is added during elongation [110]. The stretch-growth model is not an alternative to the tip-growth model but aims to provide a unified model of axonal growth. Overall, it asserts that mass addition mainly occurs on the tip in GC-mediated elongation, but is not necessarily limited to the tip when mechanical force is exogenously applied to the whole axon [60,61] or axonal growth is driven by the body mass growth. However, it is still not clear whether stretch-growth only promotes the bulk forward translocation of materials in the axon, if the local synthesis or assembly of new mass also play a role, or if stretch-growth somehow influences the rate of the axonal transport [75].

### 4.2. Exogenous Forces Affect Cytoskeletal Dynamics

The cytoskeleton, in addition to providing structural support, is also mainly responsible for modulating activity and localizing the proteins, organelles and vesicles [111]. Low mechanical forces have an effect on the actin cytoskeleton [112] and on MT dynamics [78]. Effects related to exogenous tension can involve the central and the peripheral domains of the GC [72] but also the axon shaft [113]. Depending on the mode of application of the mechanical force, it can counteract or facilitate the endogenous forces in the axonal and GC compartments, whose equilibrium influences axonal outgrowth.

Upon a certain threshold, stretching can induce F-actin filament sliding, giving rise to a relaxation of the tension [114]. Mechanical tension can also promote actin polymerization by triggering a Ca^2+^ influx via MS channels [115] or by promoting integrin-mediated point adhesion maturation [116] or counteracting the tension at the cell membrane [112]. Pita-Thomas et al. reported that a local mechanical force at the GC membrane can extend the actin cytoskeleton within the elongated portion of the filopodia and induce active lipid transport to the plasma membrane. The elongated filopodia contain polymerized actin filaments and exert retrograde forces in opposition to elongation. The retraction forces occur once the external mechanical stimulus has been removed. The author suggests that the force may lead to an increase in actin polymerization by relieving the cellular membrane tension at the filopodial tip, thus facilitating the insertion of new actin monomers [112].

**Figure 3:**
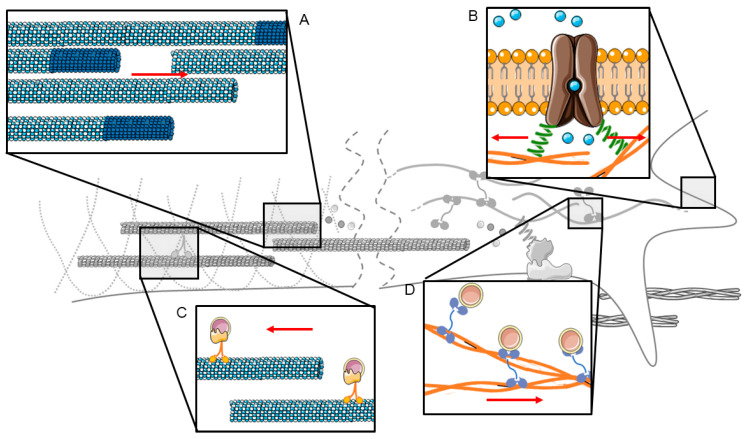
Mechanical force (red arrow) causes actin filament or MT remodeling at the GC or along the axon shaft (**A**), calcium influx by activation of MS channels (**B**), or modulation of vesicular transport along MTs (**C**) and actin filaments (**D**).

Mechanical force also influences the dynamics of MTs that form in the direction of stretch [80]. Mechanical force could influence MT sliding, as molecular motors respond to mechanically applied force [117]; MTs polymerization, as MTs act as a tension sensor [118]; and MT translocation [17]. By pulling on the GC, the MTs advance from the central domain to the peripheral domain by translocation due to actin coupling rather than by changing the polymerization/depolymerization rates [17,18]. By pulling the whole axon, stretched axons show a statistically significant increase in MT density along the entire axonal shaft from the emergency cone to the GC, but they appear normal, both in architecture and polarity [78]. Interestingly, our group showed that Nocodazole (MT-destabilizing activity), but not Paclitaxel (MT-stabilizing activity) blocked SG, highlighting that MT polymerization is crucial for sustaining SG [78]. The effect was very specific, as an inhibition of myosin II and actin polymerization had no effect on SG. The increase in MT density could help to reduce the net contractile force in the axon shaft, altering the force balance and stimulating the forward movement of the GC (Figure 3A).

### 4.3. Involvement of Exogenous Forces in Vesicular Transport

The intracellular axonal transport of vesicles, granules and organelles is a tightly and finely regulated mechanism, and its correct functioning is fundamental for cellular organization, homeostasis and survival [119]. The direct involvement of mechanical tension in axonal transport phenomena has been reported (Figure 3C,D) [54,99,120,121]. SG has been shown to decrease fast mitochondrial transport in DRG neurons [122], but to increase fast vesicle transport in *Aplysia* neurons [120]. It is not surprising, therefore, that tension can change the speed of vesicular components [123] as well as the direction, both anterogradely [72] and retrogradely [124,125]. One possible mechanism is the stretch-dependent remodeling of the cytoskeleton, which facilitates the accumulation of vesicles [100]. Tension could also influence motor activity in the axon [120], although the details of which motors are affected by tension are not known. Another mechanism is the direct manipulation of moving vesicles. For example, the magnetic labeling of vesicles with magnetic nanoparticles (MNPs) can be used to generate forces that counteract the directional movement of the lipid vesicles, causing vesicular deceleration. This can be achieved with relatively low forces in the order of tens of piconewtons [123]. The magnetic labeling of signaling endosomes halted their anterograde transport via the application of very low opposing magnetic forces (approximately 15 pN), while the bidirectional movement of mitochondria and other vesicles was unchanged, demonstrating the specificity of the mechanism [72]. Similarly, the retrograde axonal transport of magnetically labeled endosomes can be impaired with opposing magnetic forces of less than 50 pN, and stalling endosomes resume their retrograde transport suddenly after load release [124]. In addition, the authors found that this endosome capture and release mechanism could be repeated several times.

### 4.4. Exogenous Forces Induce Intracellular Calcium Influx

Mechanical stimuli can affect the behavior and function of neurons, and the development, as well as the physiology and maintenance, of neuronal networks by modulating the activity of membrane channels [126,127]. Specifically, mechanical tension controls and modulates intracellular Ca^2+^ concentrations [76]. Low-magnitude mechanical forces trigger intracellular calcium influx and modulate intracellular calcium transients [78,82,83,97]. The effects of these forces on Ca^2+^ levels are temporally confined [78,82]. Once calcium has been removed from the culture medium, neurites (including GCs) no longer respond to mechanical stress, highlighting the fundamental role of the calcium influx in the response to mechanical stimuli [97].

Franze et al. found that a local mechanical stress exceeding a defined threshold value (~274 pN µm*^−^*^2^) at the GC causes a calcium influx through MS, stretch-activated ion channels (SACs), with subsequent neurite retraction. Interestingly, the increased calcium levels propagated from the GC towards the shaft, and to other branches and the neuronal soma, disappearing after 20–30 s [97]. They also observed that applying mechanical stimuli to the neurite in other sites rather than the GC also induced calcium influx, whereas at the soma, it caused only a slight increase in Ca^2+^ levels and only in the cell body. Tay et al. found that mechanical forces ranging from 0.1 to 1 nN can induce calcium influx into cortical neural networks, increasing the magnitude and frequency of intracellular Ca^2+^ waves [82].

The mechanical stretch of the lipid membrane is likely to induce the influx of Ca^2+^ by modulating the probability of opening N-type mechanosensitive Ca^2+^ channels (Figure 3B) [83]. By investigating the spatio-temporal effect of the forces, the authors found an activation time of 5 min to observe any effect, and a rescue time of 15 min to return to a steady state [82]. Tay et al. also demonstrated that chronic stimulation with extremely low mechanical forces restores the equilibrium of N-type mechanosensitive Ca^2+^ channels, in fragile X syndrome (FXS) model neural networks, initially characterized by the downregulation of N-type mechanosensitive Ca^2+^ channels [83].

Forces may also have an effect on the intracellular calcium transients. Specifically, we found a strong attenuation of intracellular calcium transients in stretched axons, which resumed 30 min after loading removal [78]. This is consistent with the reported relationship between calcium transients and the axonal elongation rate, with a low Ca^2+^ transient associated with a rapid elongation rate and low elongation rates associated with a high Ca^2+^ transient [128].

### 4.5. Cross-Talk with Other Molecular Pathways

Discovering potential molecular pathways activated by mechanical forces is very challenging. High-throughput studies have been carried out to detail the signal mechanotransduction of exogenous forces. NGF-differentiated PC12 cells stretched for 9 h with a 1 pN force showed stretch-growth but not differential gene expression, suggesting that the primary response to force is likely to be local [79]. However, in another study exposure to similar forces (less than 5 pN) was found to induce differential gene expression, with a total of 89 dysregulated mRNAs and 43 upregulated mRNAs, which is likely due to secondary effects initiated by the force [84]. Considering that sustained axon growth is critically related to the supply of hundreds of proteins and lipids through the secretory route [129], many local mechanisms are likely to provide the mass required to sustain SG, and we are at the dawn of these studies.

Another key point that scientists are questioning is the cross-talk between chemical signals and mechanical stimuli [14]. The experimental evidence that many signaling cues modulate the intracellular force generation, mainly by slowing down the actin RF or contributing to adhesion point maturation [40,41,42,43,44,45], opens up the fascinating scenario of the force as a downstream effector of several signaling cascades. According to this view, stretched axons of hippocampal neurons do not respond to BDNF, suggesting an interference between the two pathways or a saturation effect of the neurotrophin pathway [78]. External forces are also able to guide axon growth against repulsive gradients such as those of semaphorin-3A (Sema3A) and chondroitin sulfate proteoglycans (CSPGs) [130].

Taken together, these observations make an intriguing case for the study of the application of exogenous forces as a potential therapeutic target for regeneration strategies.

## 5. Stretch-Growth: Methods and Future Therapeutic Perspectives

Despite the great interest in understanding the contribution of mechanical forces in axonal outgrowth, the implementation and translation of these research findings into preclinical or clinical settings remains challenging due to the complexity of the nervous tissue and the invasiveness of most types of approaches. Here, we briefly introduce the established methods—mainly used in cell culture or embryo models—highlighting those methodologies that have therapeutic potential.

### 5.1. Methods for the Application of Extremely Low Exogenous Forces

In the last 40 years, many technologies have been developed to mechanically stretch axons. Each technology aimed to study a specific aspect of neuronal growth (Table 1). Used for the first time by Bray in 1984 [57], force-calibrated microneedles (MNs) are the pioneer of mechanical stretching technologies. This method is based on two needles; one works as a reference, and the other one applies the calibrated force with a hydraulic micromanipulator to stretch the axon’s GC [71]. This exerts a constant axial force on the GC that can be measured from the flexure of the calibrated needle [71].

A variant of MNs—restrained bead interaction (RBI)—was then used to study CAMs using microbeads coated with antibodies [131]. The bead is directly bound to the axon membrane and restrained through a glass microneedle [26]. The range of forces exerted by the towing needle is 10^0^–10^2^ nN [26]. This amount of force impairs the RF of F-actin, causing a perturbation of the endogenous forces and moving the GC forwards [26].

The optical trap (OT) is an ingenious technology in which a microbead is held through a focalized infrared laser beam mounted on a reverse microscope [132]. It can work with a range that varies directly with the intensity of the light and inversely with the dimension of the bead [133]. It also has an optimal resolution, permitting the precise manipulation of the bead. The OT has been commonly used for measuring forces produced by molecular motors and filopodium traction forces, but is not able to exert a sufficient force to manipulate the GC [132,134,135]. Unfortunately, this technology is limited by the laser that, above a certain intensity, damages the sample.

Magnetic tweezers (MTW) work in a similar way to the OT: pairs of electromagnets manipulate para-ferromagnetic beads, which are nano- or micron-sized. Each pair of magnets modulates the movement of the beads on one axis. It is fundamental to arrange the magnets precisely. In configurations with six electromagnets, it is possible to manipulate beads in the whole sample with a constant force [130,136,137]. As with the OT, the force exerted on the sample depends on the bead dimensions, the materials composing the bead and the external field gradient. Due to its wide range of applied force and torque moment, this technology can be adapted to carry out various measurements: from filaments of DNA to whole cells [138]. MTW are usually derived from customized microscopes, and, due to the singularity of the model, this may cause a deficiency in the reproducibility of the experiments [139].

MNPs are another efficient way to manipulate neurons. Commercially used for diagnostic or drug delivery, these nanoparticles are administered as a colloidal solution [140] and are guided through magnets (dipoles). They were recently used to direct neuronal cell growth, but also to stretch neurons [66,78,79]. Generally, MNPs are no larger than 100 nm, are primarily composed of iron oxide, are coated with organic or inorganic elements, and may be conjugated with functional groups [53]. The size of MNPs facilitates internalization in neurons, allowing inward stretching on neurites [78,87].

Nanopatterned scaffolds (NSs) are a common way to study the mechanical effects of substrates on cells. They are used to mimic the extracellular environment in order to evaluate the response of neurons to substrate stiffness [141], to study the role of mechanical tension in determining the final morphology of neuronal networks [96], or to study the process of neural guidance on non-flat substrates [142].

There are also other techniques that were primarily developed for measuring forces, but they can be modified and used for applying forces. Atomic force microscopy (AFM), which is usually used to study the topography of nanostructures, has been adapted to quantify single-cell mechanical characteristics. An AFM is composed of a lever (cantilever), with a hard and sharp nanometric tip at one end. Interaction between the tip and sample causes a deflection [143]. This displacement and the rigidity of the lever are the variables that determine the force applied. To measure the mechanical properties of cells or cell compartments, the biological structure under investigation can be directly attached to the cantilever, left to adhere on the substrate, and then pulled away to break formed bonds. This technology can cover six orders of magnitude, from 10 pN to 10 µN [133].

Another experimental technique is the biomembrane force probe (BFP), which measures forces between 0.1 pN and 1 nN. A BFP is normally used to quantify the tensions of single molecular bonds. It exploits a biotinylated erythrocyte (bRBC, biotinylated red blood cell) and a microbead coated with streptavidin and the ligand of interest [144]. The red blood cell works as a spring with a variable stiffness *k*. It is directly proportional to the negative pressure derived from the aspiration of the micropipette that holds the erythrocyte. The deformation of the bRBC, due to ligand–receptor interaction, is video tracked [144]. Recently, this technique was also used to measure inter-axon adhesion [89,145].

Another way to measure traction forces that cells exert on the substrate is traction force microscopy (TFM). Cells are cultured on deformable substrates (such as hydrogels or nanowire arrays) containing some sort of detectable element that facilitates the observation of deformation [54]. On the basis of this deformation and the stiffness of the substrate, it is possible to obtain the traction force exerted by the cell toward the substrate [133]. The range of TFM varies from 10^−2^ to 10^1^ nN [146]. To study neuronal prestress on an actomyosin level, TFM was combined with AFM [147]. The measurement of forces with TFM involves minimal interaction with the sample, as force is evaluated by the deformation of pillars [148]. However, despite the high resolution obtained from TFM, the uniformity of the substrate is not suitable for simulating in vivo environments. The traction forces obtained from TFM may be considered as steady-state conditions [54].

### 5.2. New Therapeutic Perspectives

Nerve injuries trigger a complex cascade of inflammatory and pathological processes, which culminate in a scar formation, which acts to spatially contain and isolate damage [149]. Any therapeutic treatment would avoid scar formation and requires the functional reconnection of the two damaged nerve tracts [149,150]. Current practice in nerve injury treatment, for both the central and peripheral nervous systems (CNS and PNS), is based on a combination of cell transplants, the addition of neurotrophic factors/axonal guidance molecules, the elimination of inhibitory molecules, the electrical stimulation of spinal circuits, etc. [150]. Exploiting mechanical stimuli for inducing nerve regeneration, alone or in combination with other approaches, has also been investigated.

Most approaches for modulating the mechanical properties focus on reducing the stiffening of the scar. For example, the continuous administration of Taxol reduces scarring from a spinal cord injury (SCI) and promotes axonal growth at the injury site [151]. Another method is the pharmacological inhibition of the MS channels, i.e., channels that transduce the response given by nitric oxide (NO), inhibiting axonal regrowth [152].

However, the most popular mechanical approach is the use of nerve guidance conduits (NGCs) to guide nerve regeneration. Stiffness in the NGC can be used as a mechanical signal modulating cell behavior [141,153]. Mechanical inputs deriving from the stiffness of the fibers in the NGC can be delivered by modifying the concentrations and blending of polymers [154] and the porosity [155], dimensions [156,157], crystallinity [157] and anisotropy [142] of an individual fiber. Nanofibers can be used inside the NGCs to allow axons to grow through these fibers as a guide for tissue regeneration [158]. All these properties can be combined into hydrogels to give cells a similar microenvironment to the physiological one and, at the same time, a polymeric network that guides the growth direction [159].

To the best of our knowledge, the direct use of mechanical force to induce SG in the lesion site has never been investigated. This is surprising considering that recent knowledge suggests that mechanical force is perhaps the most remarkable mechanism for axonal elongation described to date [75]. One reason why the therapeutic potential of mechanical force as an inducer of axon outgrowth has been neglected for decades is the lack of methodologies that could apply research results to clinical practice.

However, recent advances in biomedical engineering and nanotechnology have opened up new perspectives. For instance, MNPs are rapidly becoming a very popular tool for stretching axons [72,78,79,82,84,87]. Thanks to their low toxicity profile, they are used in diagnostic tests as a contrast agent [160], for treating chronic anemia [161,162] and, recently, for ablation therapies in oncology [163]. Their ability to be manipulated by non-invasive magnetic fields has favored their usage for testing new therapies [164]. Furthermore, nanoparticle surfaces can be easily decorated with ligands specific for a certain cellular target [165] or modified to cross the blood–brain barrier [166]. They can also be functionalized with neurotrophins and growth factors for combined therapies [167].

**Figure 4:**
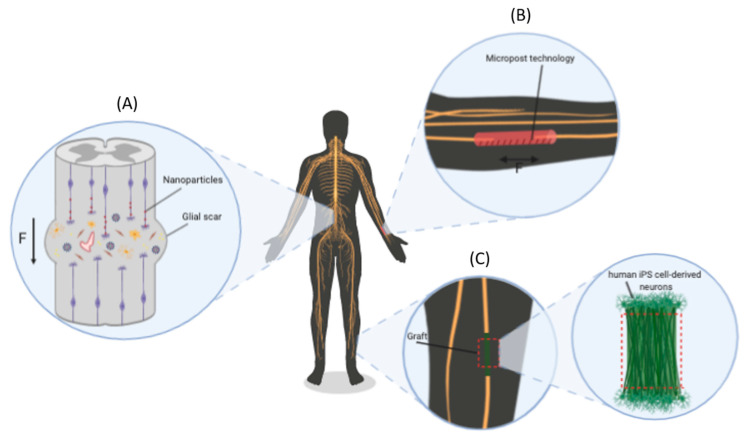
Novel therapeutic perspectives for inducing nerve regeneration through mechanical forces: (**A**) stimulation of SG of resident injured neurons via MNP labelling and their magnetic manipulation, (**B**) magnetically actuated microposts integrated in MEMs for the generation of an implantable scaffold, and (**C**) patient iPS-derived neurons stretched in vitro and used to produce implantable grafts to repair nerve gaps [173].

MNPs can be used for the magnetic labelling of entire axonal tracts. Axons of hippocampal neurons labelled with MNPs, with an iron core size of 75 nm, exposed to a magnetic field gradient of 50 Tm*^−^*^1^ were found to double their length in 48 h. MNP labelling protocols have also been tested in tissues [168,169]. Such magnetic field gradients can be easily generated and designed to have a high penetration depth in vivo. Taken together, these features make MNPs very promising and mature for pre-clinical testing (Figure 4A).

Magnetically actuated microposts are another emerging technology for the precise control of mechanotransduction in living cells. Microposts are made using a soft lithography process to fabricate an array of vertically aligned pillars of polydimethylsiloxane (PDMS) incorporating magnetic nanowires [170]. In one study [170], external magnetic fields induced a torque in the nanowires, which deflected the microposts and imparted a force on the cells attached to the array. Magnetically actuated microposts represent a potentially implantable scaffold for clinical use. Implantable scaffolds have become increasingly complex through the insertion of moving parts or conductive material to provide electrical cues, which has led to the advent of micromechanical systems (MEMs) [171]. Magnetic microposts can be easily integrated into MEMs, and, depending on the miniaturization of the pillars, forces ranging from a few piconewtons to hundreds of nanonewtons can be applied [172] (Figure 4B).

Another approach, originally proposed by D.H. Smith, consists of developing transplantable nervous tissue constructs to repair nerve gaps. Specifically, thanks to the ability of axon tracts from DRG neurons to undergo extreme SG and the integrity of the stretched cultures once removed from the in vitro environment, stretched neurons can be used to produce an in vitro “living bridge” for transplantation in the lesion site as an alternative to the acellular grafts and NGCs [4]. Interestingly, the combination of the potentiality of SG with the emerging discoveries in stem cells could make it feasible to derive transplantable nervous tissue constructs directly from adult patient-derived induced pluripotent stem cells (iPS cells) after neuronal differentiation [173] (Figure 4C).

## 6. Conclusions

This paper attempts to provide an understanding of how exogenous forces can be used to gain control of axonal outgrowth, speculating about technologies that may have future therapeutic relevance. Many stages during neuron growth and development seem to be modulated by the application of exogenous forces. Forces acting over different magnitudes and time scales (including those mimicking endogenous forces acting at low magnitudes and over long time scales) can induce axonal growth. Exogenous forces also seem to replicate the effect of the endogenous ones, acting on cytoskeleton remodeling, axonal transport modulation and the activation of MS channels. Besides, fundamental questions regarding the local mechanisms triggered by force application are not fully elucidated, and novel mechanistic studies are urgently required to address this point. Another related question is whether the application of mechanical force may be exploited as an additional level of control of axonal growth for implementing regenerative strategies. Recently, technological progress has enabled the induction of SG by many different technologies. Some of them have clinical potential. However, to date, none of them are in the pre-clinical stage. To reach this stage of development, further studies are needed, although current results are already very promising. At present, we are only beginning to understand how endogenous mechanical forces take place in the nervous system and how exogenous ones can be used in vivo or ex vivo to accelerate repair mechanisms by mimicking developmental processes.

## Figures and Tables

**Table 1:** Main methods for the application of exogenous forces. MN (force-calibrated microneedles), RBI (restrained-bead interaction), AFM (atomic force microscopy), BFP (biomembrane force probe), OT (optical trap), MTW (magnetic tweezers), MNP (magnetic nanoparticles), TFM (traction force microscopy), MEM (micromechanical system), SCG (superior cervical ganglion), P (post-natal), E (embryonal), NS (nanopatterned scaffold). Quantification gives pure force (pN), stress (pN µm*^−^*^2^), stretch rate (µm h*^−^*^1^) or strain (%), based on the data present in the mentioned articles. N/A = data not available.

Methods	Quantification	Biological Model	Effect	Ref.
MN	N/A	Chick DRG (E7–E12)	Axon branching	[92]
	40–1000 µm h^−1^	Chick DRG (E10–E12)	Neurite initiation/axon elongation/axon branching	[57]
	10^2^–10^3^ pN	Chick DRG (E12)	GC-mediated axonal elongation	[60]
	10^2^–10^3^ pN	PC12 cells + Chick DRG (E10–E12)	Axon elongation/axon pruning	[69]
	10^0^–10^3^ pN	Chick DRG (E10–E12)	Neurite initiation/neurite elongation	[61]
	10^1^–10^3^ pN	Chick sensory neurons (E7–E8)	Neurite initiation/axon elongation	[59]
	10^2^–10^4^ pN	PC12 cells	Neurite initiation/axon elongation	[58]
	~10^2^ pN	Rat hippocampal neurons (E18–E19)	Neurite to axon specification	[65]
	10^3^ pN	Rat hippocampal neurons (E18)	GC motility + neurite extension	[135]
	10^−4^–10^3^ pN	Rat RGC (E13–P8)	Axon elongation	[77]
	10^2^–10^5^ pN	*Aplysia* bag cell neurons	GC traction force + cytoskeletal dynamics	[26]
RBI	190–310 µm h^−1^	*Aplysia* bag cell neurons	GC motility + cytoskeletal dynamics	[131]
	N/A	*Aplysia* bag cell neurons	Regulation of MT behavior during neuronal growth	[18]
	2.5–100 µm h^−1^	Chick DRG (E12)	Axon elongation	[71]
AFM	~10^3^ pN	Fly embryo motoneurons	Synaptic vesicle accumulation	[100]
	10^2^–10^5^ pN	*Aplysia* bag cell neurons	GC traction force + cytoskeletal dynamics	[26]
	~10^2^ pN	Rat sensory neurons (E17–E18)	Neurite initiation/axon elongation/network formation	[63]
BFP	10^1^–10^3^ pN	Mouse olfactory sensory neurons (E13.5)	Axon fasciculation/defasciculation	[89]
OT	~10^0^ pN	Rat hippocampal neurons (E18)	GC motility + neurite extension	[135]
	10^−1^ pN	*Carassius auratus* retinal ganglion cells	Axon orientation	[86]
MTW	10^0^–10^3^ pN	Chick sensory neuron (E7–E8)	Neurite initiation/axon elongation	[62]
	10^0^–10^2^ pN	Mouse cortical neurons (E14)	Mechanochemical axon elongation	[130]
	5–400 pN µm^−2^	Rat cortical neurons (E18)	Study of rheological properties	[137]
MNP	10^1^–10^2^ pN	Rat RGC (E20–P8)	Axon elongation	[72]
	~10^0^–10^1^ pN	Neuron-like PC12 cells	Axon orientation	[87]
	10^0^–10^3^ pN	Rat cortical neurons (E18)	Axon specification/axon orientation	[66]
	~10^0^–10^1^ pN	Rat RGC (P0-P4)	Directional filopodia elongation/actin cytoskeleton polymerization	[112]
	10^2^–10^3^ pN	Rat cortical neurons (E18)	Axon elongation/intracellular Ca^2+^ influx induction	[82]
	10^2^–10^3^ pN	Rat cortical neurons (E18)	Intracellular Ca^2+^ influx induction	[83]
	~10^0^–10^2^ pN	Rat cortical neurons (E18)	Vesicle speed alteration	[123]
	$\sim 10^{0}$ pN	PC12 cells + SH-SY5Y	Axon elongation	[79]
	~10^0^–10^1^ pN	Rat DRG neurons (E18)	Axonal transport alteration	[125]
	$\sim 10^{0}$ pN	PC12 cells + rat DRG (P1–3)	Axon elongation	[84]
	10^0^–10^1^ pN	Mouse hippocampal neurons (P0–P1)	Axon elongation/branching/axon excitability	[78]
TFM	10–2000pN µm^−2^	NG108-15 cells	Axon branching/axon pruning	[97]
	~10^3^ pN	Adult mouse DRG + adult mouse SCG	GC pathfinding	[146]
MEM	84 µm h^−1^	Rat cortical neurons + differentiated human neurons from NT2 cell line	Axon elongation	[73]
	0.2–2%	Rat DRG (E15)	Axonal elongation	[75]
	42–250 µm h^−1^	Rat DRG (E15)	Mitochondrial transport alteration	[122]
	0–20%	Drosophila embryo motor neurons + *Aplysia* neurons	Synaptic vesicle accumulation	[99,120,121]
	2.5–4.2%	One-month mouse brain slices	Axon excitability	[102]
	5–52%	Rat DRG (E16)	Axonal elongation	[76]
	7–28%	Rat cortical neurons (E18–E19)	Axon elongation/axon branching/axon orientation	[80]
	4.2–83.3 µm h^−1^	Rat DRG (E16) + rat motor neurons (E16)	Axon elongation/axon fasciculation	[81]
NS	N/A	Adult locust frontal ganglions	Axon branching/axon pruning	[96]
	N/A	Mouse hippocampal neurons P1 + PC12 cells	Neuronal network activity	[141]

## Abbreviations

AFM	Atomic force microscopy
BDNF	Brain-derived neurotrophic factor
BFP	Biomembrane force probe
bRBC	Biotinylated red blood cell
C	Central
CAMs	Cell adhesion molecules
CNS	Central nervous system
CSPG	Chondroitin sulfate proteoglycans
DIV	Days in vitro
DRG	Dorsal root ganglia
ECM	Extracellular matrix
FA	Focal adhesion
FAK	Focal adhesion kinase
FXS	Fragile X syndrome
GC	Growth cone
iPS	Induced pluripotent stem cell
MAPs	MT-associated proteins
MEMs	Micromechanical systems
MNPs	Magnetic nanoparticles
MNs	Microneedles
MS	Mechanosensitive
MT	Microtubule
MTW	Magnetic tweezers
NGC	Nerve guidance conduit
NGF	Nerve growth factor
NMII	Non-muscle myosin II
NO	Nitric oxide
NSs	Nanopatterned scaffolds
OT	Optical trap
P	Peripheral
PAK	P-21 activated kinase
PDMS	Polydimethylsiloxane
PKC	Protein kinase C
PNS	Peripheral nervous system
RBI	Restrained bead interaction
RF	Retrograde flow
RGC	Retinal ganglion cells
ROCK	Rho-associated protein kinase
RPTP-alpha	Protein-tyrosine phosphatase alpha
SACs	Stretch-activated ion channels
SCI	Spinal cord injury
SEMA3A	Semaphorin-3A
sEPSCs	Spontaneous excitatory postsynaptic currents
SG	Stretch-growth
T	Transition
TFM	Traction force microscopy
